# Exploring Socio-Economic Characteristics Associated With Having a Live Birth Using Assisted Reproductive Technology Compared to Conceiving Naturally: Evidence From the UK Household Longitudinal Survey 2009-2022

**DOI:** 10.34172/ijhpm.9268

**Published:** 2026-07-19

**Authors:** Heather Brown, Sophie Guthmuller

**Affiliations:** ^1^Division of Health Research, Lancaster University, Lancaster, UK.; ^2^Health Economics and Policy Group, Vienna University of Economics and Business, Wien, Austria.

**Keywords:** In Vitro Fertilisation, Assisted Reproductive Technology, Socio-Economic Inequalities, Reproductive Autonomy, Health Care Access, England

## Abstract

**Background:**

In England, the National Institute for Health and Care Excellence (NICE) guidance recommends that women under 43 years old are offered 3 cycles of in vitro fertilisation (IVF) on the publicly funded healthcare system if they have not conceived after two years. However, regional commissioners decide the funding allocated for IVF. Our study aims to assess socio-economic and regional factors associated with having a live birth after using IVF over time to determine if decreases in funding for IVF increase regional inequalities in live birth success rates.

**Methods:**

We use Understanding Society Survey data from 2009-2022 employing a random effects probit model to estimate the determinants associated with a live birth via IVF among women having given birth in the previous year. Next, we estimate a random effects probit model to compare the determinants of live birth from IVF between 2009-2018 and 2019-2022 to assess the impact of unequal decreases in funding across different regions of England.

**Results:**

Between 2009-2022, living in London, being older, in employment, degree level or higher education, and owning/mortgage home are positively and statistically significantly associated with a live birth using IVF. Comparing 2009-2018, to 2019-2022 with less generous funding we find that in Yorkshire and Humber, there is a decrease in women successfully conceiving using IVF. Married/cohabiting women are three percentage points more likely to have a live birth with IVF compared to single women in 2019-2022 compared to 2009-2018. Women living in a middle deprivation quintile area (3) are three percentage points more likely to have a live birth from IVF in 2019-2022 compared to 2009-2018.

**Conclusion:**

Lack of individual resources in conjunction with regional difference in in IVF funding contribute to inequalities in reproductive autonomy by reducing opportunities to access IVF.

## Background

**Key Messages**

**Implications for policy makers**

- Our research provides evidence on inequalities in live birth outcomes from in vitro fertilisation (IVF) in England.
- Inequalities in IVF outcomes by socio-economic status have increased when health budgets became constrained stemming from the 2008 financial crisis and the COVID-19 pandemic.
- To support the implementation of the Women’s Health Strategy and goal of increasing access and ensuring equal access across England funding for assisted reproductive technology (ART) such as IVF should be earmarked so that commissioners cannot reduce funding or apply additional criteria for IVF in different Integrated Care Boards.

**Implications for the public**

There are policy recommendations in England that all women under the age of 43 who are having difficulty conceiving after 2 years and do not have any children should have access to 3 cycles of in vitro fertilisation (IVF) provided free at point of use by the National Health Service (NHS). However, the ultimate decision on if IVF is funded and how many cycles of IVF are funded at the regional level is determined by regional funding commissioners at Integrated Care Boards (ICB). Funding constraints have reduced or eliminated in some regions publicly funded IVF. We explore using the Understanding Society Survey what are the characteristics of women who had a baby using IVF and if these change when there is less funding for the health service. We find that where a person lives, employment, home ownership, and educational attainment, are significant determinants of a successful birth using IVF.

Over the last 25 years there has been growing recognition of the global importance of enshrining in health guidelines and recommendations on women’s right to reproductive healthcare including accessing assisted reproductive technologies such as in vitro fertilisation (IVF).^1^ The Convention on the Elimination of All Forms of Discrimination Against Women (CEDAW) was originally adopted in 1979.^2^ It outlined what is considered discrimination against women and how it can be stopped. In the 18th session of the CEDAW in 1998, the rights to reproductive and sexual health were discussed. This included the right to reproductive choice to conceive, to end an unwanted pregnancy, and choose a preferred method of family planning and contraception. Most importantly, for health economics the equitable distribution of resources for women’s health needs, which are often the first services to be cut when budgets are reduced, was outlined.^1,3^

 In the UK, the National Institute for Health and Care Excellence (NICE) develops guidance for health practitioners to support evidence-based decision-making.^4^ NICE guidelines recommend that women under the age of 43 are offered 3 cycles of IVF if they have not conceived after two years of regular vaginal intercourse or 12 cycles of artificial insemination (including 6 or more by intrauterine insemination).^5^ However, the final decision regarding if treatment is offered by the National Health Service (NHS) is made at the local level by Integrate Care Boards (previously Clinical Commissioning Groups, who are responsible for the planning and funding of health services locally.^6^ This means that local areas can make different decisions on how much public funding to allocate to IVF resulting in regional differences in public access. Nationally, the proportion of IVF funded by the NHS has been in decline in all regions and saw drops during and after the COVID-19 pandemic, as illustrated in Figure. On average 40% of IVF cycles were NHS funded in 2012 whilst it was 24% in 2022 in England, and regional differences persisted over the period. The North East of England has the highest level of publicly funded IVF whereas the East of England and London have the lowest levels of publicly funded IVF.

**Figure:**
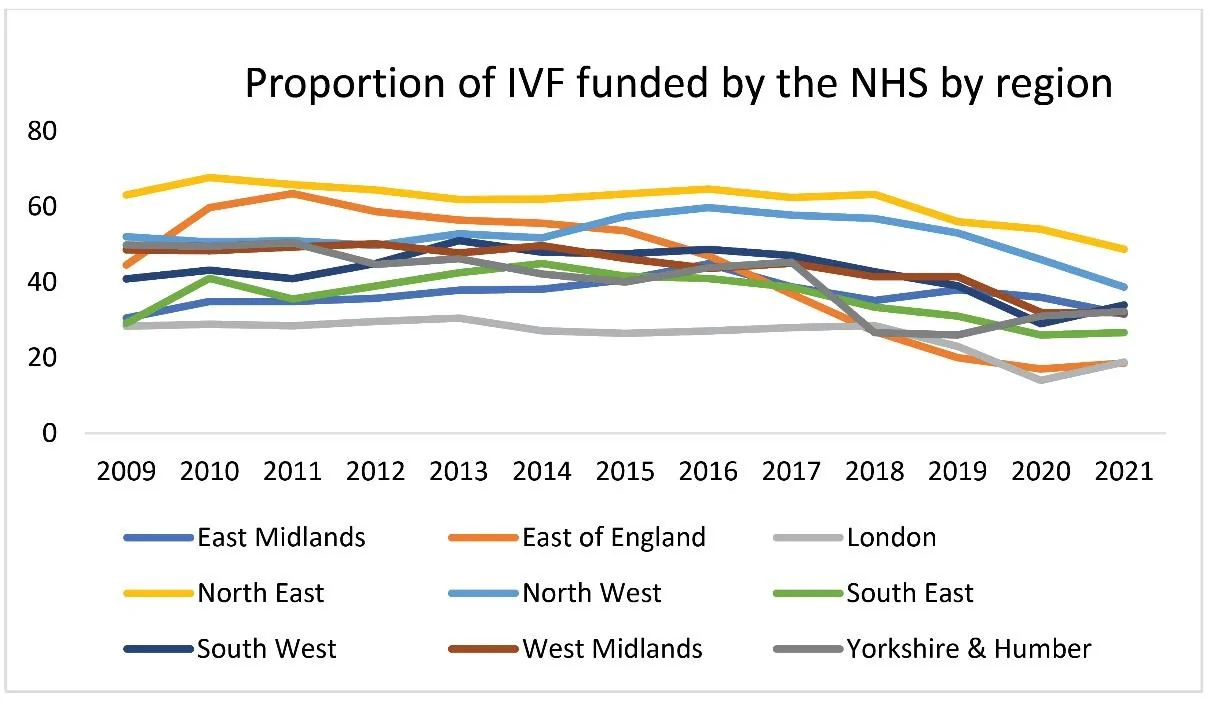
Proportion of In Vitro Fertilisation Funded by the National Health Service by Region. Note: own figure based on data from Human Fertilisation Embryology Authority. Abbreviations: IVF, in vitro fertilisation; NHS, National Health Service.

 Because of the high costs of medical technology, such as IVF, and with pressures on budgets, this is a service that commissioners can rationalise decommissioning to save costs.^7^ In the UK in 2019, the percentage of IVF treatments that led to a live birth was 35% for those under 35, 25% for women 35-37, 19% for women 37-39, 11% for women aged 40-42, 5% for women 43-44, and 4% for women over 44.^8^ Evidence from the UK suggests that on average 6 cycles of IVF have the highest rate of success (65%) with variations by age and treatment type.^8^ This suggests that most women who conceive will have to privately fund multiple cycles of IVF.^9^ Women who cannot afford private IVF cycles will not be able to realise their right to reproductive choice to conceive. Women with lower incomes and less access to resources to fund private cycles living in areas that have seen larger declines in publicly funded cycles such as the East of England, South East, and Yorkshire and Humber may have a lower likelihood of having a live birth from IVF increasing inequalities. Qualitative research by Hamper and Perrotta^10^ found implications for the equitable distribution of resources based upon need.

 Fertility problems and help seeking behaviour may vary by socio-economic status, leading to inequalities in birth outcomes. There is a number of studies from across Organisation for the Economic Co-operation and Development countries investigating the relationship between socio-economic status, the use of IVF, and the health system.

###  Socio-Economic Status

 A study from Finland between 1996-1998 found that the majority of the allocation of public expenditure for IVF was for younger women from higher socio-economic groups.^11^ A recent study from Denmark^12^ found an increased odds of receiving assisted reproductive technology (ART) treatment for women in either the public or private sector, for women with higher levels of educational attainment, and women who were employed. Interestingly, women with higher incomes were more likely to access ART from the public sector than those on lower incomes. Using linked data on area level deprivation (a multidimensional measure assessing factors at the area level such as employment, educational attainment, health status, housing quality, transport, and crime) and number of clinics and IVF cycles provided at each clinic from the Human Fertilization and Embryology Authority data in the UK, Jones et al^13^ found that women living in less deprived areas had greater access to IVF. A recent paper from 2024 including data from Denmark, France, Spain, the UK, and the US found evidence of an educational gradient in ART birth that is steeper among countries with less favourable ART access policies.^14^ Chanfreau et al^15^ showed that the contribution of ART on fertility rate may be small overall, but considering the specific need and population that ART aims to cover, use of ART contributed significantly to reduce involuntary childlessness, specifically among higher socio-economic groups and older women in Norway.

 At the same time, age-specific rates of infertility vary by area level deprivation. Women living in deprived areas under 25 are 5.6 times more likely to report fertility issues than women living in less deprived areas, with deprivation measured by the Townsend index.^16^ There is evidence of a socio-economic gradient in women seeking help with infertility.^17^ Decreasing public funding to IVF decreases access.^18^

 More research is needed on fertility treatment outcomes and their link with socio-economic status, as recently summarised in a literature review.^19^ Previous works considered only one or some of the dimensions (income, education, occupation, family situation).^11,20-25^ There is also less evidence on other key social determinants of health and fertility outcomes, such as housing tenure type, ethnicity, and area-level deprivation in Europe. Evidence from the US, found for instance that Black and Hispanic women are associated with less ART birth than white women.^24^ Fathers of ART birth are also more likely white and high educated compared to fathers of natural birth.^26^

###  Health System

 A study of medical files in France in the 1990s^20^ showed that the structure of the healthcare system exacerbated inequalities. Women from different socio-economic backgrounds had different experiences of the medical system leading them to making different decisions related to the risks of the treatment not being successful.

 Health system financing for IVF is an important determinant of successful birth outcomes. A study in Australia found that the reduction in public funding for fertility treatment reduced significantly the number of ART birth overall.^18^

 Wider spending outside of the health services may also influence fertility decisions leading to inequities in birth outcomes. Sochas and Chanfreau^27^ found that reductions in local government funding associated with the austerity period in the UK reduced the likelihood of women with low incomes having a second child by 9.1% compared to women with higher incomes.

 The aim of this paper is to identify the socio-demographic characteristics of women who successfully gave birth using IVF and compare them with women who conceived without using ART in the last twelve months. The second aim of the paper is to estimate if the magnitude and significance of the socio-demographic characteristics of women who successfully gave birth using IVF changes between a more generous funding regime (2009-2018) and a more restrictive one (2019-2022). These findings can be used to inform policy to support women’s reproductive autonomy.

 Our study contributes to the literature by identifying how sociodemographic and health system design and delivery may contribute inequalities in reproductive healthcare and outcomes.

## Methods

 We follow the STrengthening the Reporting of OBservational studies in Epidemiology (STROBE) reporting guidelines for observational studies.

###  Data

 The data comes from Waves 1-13 (2009-2022) of the Understanding Society Survey.^28^ The Understanding Society Survey is a longitudinal household survey of approximately 40 000 households where household members aged 16 and above are interviewed annually. Participants were selected using a two-stage sampling procedure. Primary selection is from postcode which were then grouped into larger geographical areas such as region to ensure a nationally representative selection of households.^28^ Information is collected on a range of topic areas, including health, work, education, income, and family, using a range of both subjective and objective measures. We restrict our sample to women between the ages of 18-50 who have given birth to a live child within the past year. We perform a complete case analysis with 8130 observations over the sample period. We do not employ study weights as we are looking at a specific sub-set of the population – women who have given birth to a child within the past year. Thus, our sample is not nationally representative. Women can leave and re-enter the survey if they have multiple children (eg, have given birth within the past year), because of this entry/exit we do not consider attrition in the wider eligible population.

 Our main outcome variable is a binary variable which equals one if a woman reports conceiving using IVF and zero if she reports giving birth without using any medical technologies.

 We include a range of explanatory variables to explore what socio-demographic characteristics are associated with an increased likelihood of conceiving using ART compared to not requiring a medical intervention. We hypothesize that those with greater access to resources are more likely to have given birth using IVF. Women with more resources are more likely to be able to afford privately funded multiple rounds of IVF which increases the likelihood of a successful live birth.^9^ We do not assume that access is impacted by region, but that region may be one factor that impacts on the likelihood of having a live birth. We also explore the intersectionality of region and other socio-economic characteristics.

 In our estimation model, we include a continuous variable for age. We include a variable indicating the proportion of IVF funded by the NHS annually in the region where the woman lives. We include a binary variable that equals one if the woman lives in an urban area and equals zero if she lives in a rural area, as well as a binary variable that equals one if the woman is married or cohabiting and equals zero otherwise. We include a categorical variable for area-level deprivation measured using the Index of Multiple Deprivation (IMD). IMD combines information across seven domains—(1) income; (2) employment; (3) education, skills, and training; (4) health and disability; (5) crime; (6) barriers to housing and services; and (7) environment—to produce an overall deprivation score for small geographical areas in England. IMD is represented as a categorical variable divided into quintiles, with one being the most deprived and five being the least deprived.^29^ We also include a continuous variable for gross monthly pay for the woman, which is equal to zero if the woman is not in employment. We include two binary variables for type of housing tenancy: one is a variable which equals one if the woman lives in a socially rented house and is equal to zero otherwise; the other is a variable which equals one if the woman lives in a privately rented house and is equal to zero otherwise. The reference category for both housing tenancy variables is if a woman lives in a house that is owned outright or she has a mortgage. We include a binary variable which equals one if the person is employed (full-time, part-time, or self-employed) and is equal to zero otherwise. Finally, we include a binary variable for ethnicity which equals one if a woman reports being of White British or Irish ethnicity and zero if she reports any other ethnicity. We include two measures of health status^30^: (1) 12-item Short Form Survey (SF-12) physical health and (2) SF-12 mental health. It is a self-reported outcome measure to assess health in the general population. It assesses health across the following 8 areas: (1) limitations in physical activities because of health problems; (2) limitations in social activities because of physical or emotional problems; (3) limitations in usual role activities because of physical health problems; (4) bodily pain; (5) general mental health (psychological distress and well-being); (6) limitations in usual role activities because of emotional problems; (7) vitality (energy and tiredness); and (8) how the person feels about their own health. A person’s score across these eight different areas is summed together to create a total score which takes values between 0 for the worst possible health to 100 for the best possible health. We include eight binary variables for the regions of England with the reference category “Greater London” for each of these binary variables.

###  Estimation Strategy

 To address our first aim of estimating the socio-economic characteristics associated with having a live birth conceived via IVF compared to a live birth conceived naturally we estimated a random effects probit model that accounts for time and individual heterogeneity estimated across the whole study period (2009-2022). We employ a probit model as when controlling for random effects within individuals over time, the normal distribution assumption of the probit model means that we can fully categorise the joint distribution of the underlying latent variables.^31^ We account for unobserved time varying differences between individuals, but do not employ a fixed effects model as a Hausman test suggests that a random effects model is more efficient. Formally the model is presented as:


(1) $$Y_{it}^{*}=\alpha +X_{it}\beta +\varepsilon_{it}+\mu_{i}$$

(2) $$Y_{it}^{*}=\left\{\begin{array}{l} 1\;if\;Y_{it}^{*}\geq 0\\ 0\;if\;Y_{it}^{*}<0 \end{array}\right.$$

Where $Y_{it}^{*}$ is a binary variable which is equal to one if the woman *(i) *gave birth to a live child in the past year *(t)* using IVF and is equal zero if a woman *(i) *gave birth to a live child without the use of reproductive technologies in the past year *(t)*. The vector *X_it_* includes woman’s *(i)* socio-demographic information such as educational attainment, marital status, area level deprivation, income, housing tenancy type, age and health status over the study period *(t)*. The error term is comprised of a random error component, *ε_it_* and an individual specific error component, *μ_i_*. Equation (2) shows the underlying latent distribution of the outcome variable.

 Next, for our second objective to explore if inequalities have widened with a decrease in funding available for IVF, we estimate a before/after probit model. We compare those who had a live birth between 2009 and 2018 when there was more generous publicly available funding for IVF to 2019-2022 when the funding for IVF was reduced. Reductions in funding was heterogenous between regions, and we explore if and how this heterogeneity in funding at the regional level was associated with the likelihood of conceiving using IVF. The model controls for time and individual heterogeneity.

 This equation is presented as:


(3) $$Y_{it}^{*}=\alpha +\beta Post_{t}\times X_{it}+\varepsilon_{it}+\mu_{i}$$

Where $Y_{it}^{*}$ , *X_it_*, *ε_it_*, and *μ_i_* are defined above. In equation (3), *Post_t_* is an indicator variable for the period where most regions in the UK saw a reduction in funding (2019-2022) and is equal to zero for the time between 2009-2018. Post is interacted with the explanatory variables in the matrix *X_it_* to identify if there is a change in the slope of the probability of a change in the explanatory variable leading to a change in the outcome variable in the two time periods.

## Results

###  Univariate Analysis

 We start by presenting some descriptive statistics for our sample in Table 1. We have data on 10 535 women who have given birth to a baby in the previous year of these women, 10 219 did not use ART and 316 used IVF. Means (percentages for binary variables), standard deviations, and sample size are shown. We estimate the mean difference in continuous variables using student *t* tests and employ Wilcox rank-sum tests for binary variables comparing characteristics for those who conceived without using ART and those who use IVF.

**Table 1:** Descriptive Statistics

**Used IVF to Get Pregnant**	**Yes**			**No**			Difference*	
**Variables**	**Mean**	**(SD)**	**n**	**Mean**	**(SD)**	**n**		***P*** ** Value**
Age	‎34.56‎	‎(5.58)‎	‎316‎	‎30.86‎	‎(5.78)‎	‎10 219‎	‎3.71‎	<.001
IMD	‎3.29‎	‎(1.36)‎	‎258‎	‎2.74‎	‎(1.41)‎	‎8140‎	‎0.54‎	<.001
Employed	‎0.65‎	‎(0.48)‎	‎316‎	‎0.45‎	‎(0.50)‎	‎10 215‎		<.001
Educational attainment								
Degree or higher	‎0.67‎	‎(0.47)‎	‎267‎	‎0.46‎	‎(0.50)‎	‎10 219‎		<.001
Some higher qualifications	‎0.11‎	‎(0.31)‎	‎316‎	‎0.13‎	‎(0.34)‎	‎10 219‎		.385
Basic qualifications	‎0.11‎	‎(0.31)‎	‎316‎	‎0.22‎	‎(0.41)‎	‎10 219‎		<.001
No qualifications	‎0.01‎	‎(0.08)‎	‎316‎	‎0.01‎	‎(0.10)‎	‎10 219‎		.597
Marital status								
Married/Cohabiting	‎0.95‎	‎(0.21)‎	‎316‎	‎0.82‎	‎(0.38)‎	10 195		<.001
Single	‎0.03‎	‎(0.19)‎	‎316‎	‎0.15‎	‎(0.36)‎	10 195		<.001
Urban	‎0.79‎	‎(0.40)‎	‎314‎	‎0.81‎	‎(0.39)‎	10 192		.554
Mean gross pay at last pay cheque	5929.04	(13 636.54)	316	3341	(9629)	10 219	-2587.21	<.001
SF-12 physical	‎50.62‎	‎(9.50)‎	‎301‎	‎51.92‎	‎(8.63)‎	‎9363‎	‎1.35‎	.079
SF-12 mental	‎48.38‎	‎(9.45)‎	‎301‎	‎47.07‎	‎(10.31)‎	‎9363‎	‎-1.31‎	<.05
Housing status								
Own house/mortgage	‎0.79‎	‎(0.41)‎	‎315‎	‎0.57‎	‎(0.49)‎	‎10 153‎		<.001
Social rent	‎0.05‎	‎(0.22)‎	‎315‎	‎0.22‎	‎(0.41)‎	‎10 153‎		<.001
Private rent	‎0.12‎	‎(0.32)‎	‎315‎	‎0.15‎	‎(0.36)‎	‎10 153‎		<.001
White British/Irish	‎0.76‎	‎(0.43)‎	‎316‎	‎0.74‎	‎(0.44)‎	‎10 187‎		.601
Regions								
North East	‎0.03‎	‎(0.17)‎	‎316‎	‎0.03‎	‎(0.17)‎	10 219		.862
North West	‎0.09‎	‎(0.29)‎	‎316‎	‎0.10‎	‎(0.31)‎	10 219		.472
Yorkshire & Humber	‎0.05‎	‎(0.22)‎	‎316‎	‎0.09‎	‎(0.28)‎	10 219		<.05
East Midlands	‎0.05‎	‎(0.22)‎	‎316‎	‎0.08‎	‎(0.27)‎	10 219		.105
West Midlands	‎0.06‎	‎(0.24)‎	‎316‎	‎0.09‎	‎(0.28)‎	10 219		.081
East of England	‎0.11‎	‎(0.32)‎	‎316‎	‎0.09‎	‎(0.28)‎	10 219		.107
Greater London	‎0.15‎	‎(0.36)‎	‎316‎	‎0.14‎	‎(0.34)‎	10 219		.337
South East	‎0.17‎	‎(0.38)‎	‎316‎	‎0.11‎	‎(0.32)‎	10 219		<.05
South West	‎0.08‎	‎(0.28)‎	‎316‎	‎0.07‎	‎(0.26)‎	10 219		.380

Abbreviations: IVF, in vitro fertilisation; SF-12, 12-item Short Form Survey; IMD, Index of Multiple Deprivation; SD, standard deviation. Notes: Descriptive statistics of the variables used in the analysis are shown. * Differences in mean are reported for continuous variables. *P* values of the mean difference in continuous variables using student *t* tests and Wilcox rank-sum tests for binary variables comparing characteristics for those who conceived using IVF and those who did not use IVF to get pregnant are displayed.

 There is a statistically significant difference of 3.71 years in age for women who conceive without ART (mean of 30 years old) to 34 years old for those who conceive using IVF. Women who conceive using IVF live in areas of less deprivation, which is statistically significantly different compared to women who conceive without ART. Approximately 47% of those who conceive without ART are educated to the degree level or higher, compared to 67% of those using IVF being educated to the degree level or higher. This difference in educational attainment is statistically significant. Those who conceive using IVF compared to those who do not conceive using ART are statistically significantly less likely to have basic qualifications. Approximately 95% of women using IVF are married compared to 82% who do not use ART; this difference is statistically significant. There is no statistically significant difference by urbanity between women who conceive using IVF and those who do not. There is a statistically significant mean difference in monthly gross pay, with women conceiving using IVF earning on average £2587.21 more than those who did not use IVF. Women who conceive without IVF have a SF-12 physical health score of 51.92 compared to those who use IVF who have a mean SF-12 physical health score of 50.62. This difference is not statistically significant at *P* < .05. However, there is a statistically significant difference in mental health as measured by SF-12 at *P* < .05, with women who conceive without using IVF reporting a mean SF-12 mental health score of 47.07 and those who conceive using IVF reporting a mean SF-12 mental health score of 48.38. Approximately 79% of women who conceive using IVF own outright or have a mortgage on their home compared to 57% of women who conceive without using ART. This difference is statistically significant. There is also a statistically significant difference between the percentage of women who privately rent and socially rent by conception type. There is no statistically significant difference in conception type by ethnicity. For the majority of regions, there is no statistically significant difference by conception type, with the exception of the South East and Yorkshire and Humber.

###  Multivariable Analysis

 Next, in Table 2 we show the results from our main estimation model, a random effects probit model. Marginal effects are shown. Marginal effects show the change in probability of going from zero to one for the outcome variable for a one-unit change for continuous variables or moving from zero to one for binary variables, which are interpreted as percentage points. Older women are statistically more likely to conceive with IVF than without ART, but the marginal effect is very small. Compared to women living in London, women living in Yorkshire and Humber are 3 percentage points less likely to conceive using IVF and women living in the East and West Midlands are 2 percentage points less likely to conceive using IVF. Married or cohabiting women are 2 percentage points more likely to conceive using IVF. Compared to women who own their own home or have a mortgage on their home, women living in social housing are 3 percentage points less likely to conceive using IVF and those in private rented accommodation are 1 percentage point less likely to conceive using IVF (but this is not statistically significant). Women in employment are 2 percentage points more likely to conceive using IVF than women who are unemployed or not in the labour force. Those with worse physical health are statistically significantly less likely to conceive using IVF than those with better health, but the marginal effect is very small. Women educated to the degree level are 2 percentage points more likely to conceive using IVF than those with no qualifications.

**Table 2:** Random Effects Probit Model of Determinants of Conceiving by In Vitro Fertilisation (Live Birth): 2009-2022

**Variables**	**Marginal Effects**	**(SE)**
Age	0.00***	(0.00)
Regions		
North East	‎-0.01‎	‎(0.01)‎
North West	‎-0.01‎	‎(0.01)‎
Yorkshire & Humber	‎-0.02**‎	‎(0.01)‎
East Midlands	‎-0.02**‎	‎(0.01)‎
West Midlands	‎-0.01‎	‎(0.01)‎
East of England	‎-0.00‎	‎(0.01)‎
Greater London	Ref.‎	Ref.‎
South East	‎0.00‎	‎(0.01)‎
South West	‎-0.00‎	‎(0.01)‎
Marital status		
Married/Cohabiting	0.02*	(0.01)
Single	Ref.	Ref.
Urban	0.00	(0.01)
IMD		
Decile 1	Ref.	Ref.
Decile 2	‎-0.01‎	‎(0.01)‎
Decile 3	‎0.01‎	‎(0.01)‎
Decile 4	‎0.00‎	‎(0.01)‎
Decile 5	‎0.01‎	‎(0.01)‎
Mean gross pay at last pay cheque	‎0.00‎	‎(0.00)‎
Housing status		
Own house/mortgage	Ref.	Ref.
Social rent	‎-0.03***‎	‎(0.01)‎
Private rent	‎-0.01‎	‎(0.01)‎
Employed	0.02***	(0.00)
White British/Irish	0.01	(0.01)
SF-12 physical	-0.00***	(0.00)
SF-12 mental	-0.00	(0.00)
Education attainment		
Degree or higher	‎0.02*‎	‎(0.01)‎
Some higher education	‎0.01‎	‎(0.01)‎
Basic education	‎0.01‎	‎(0.01)‎
No qualifications	Ref.‎	Ref.‎
Observations	6863	

Abbreviation: SF-12, 12-item Short Form Survey; SE, standard error; IMD, Index of Multiple Deprivation. Notes: Marginal effects are shown. Standard errors in parentheses. ***P < .01, ** *P* < .05, * *P* < .1.

 None of the other marginal effects are statistically significant. However, compared to London all other areas are less likely to conceive using IVF. Women living in less deprived areas are more likely to conceive using IVF as were women with higher levels of education. The marginal effect on gross pay at last pay cheque and mental health as measured by the SF-12 are both zero.

Table 3 shows the results comparing the association of individuals determinants and the likelihood of having a live birth via IVF between 2012-2018 and 2019-2022 after funding was cut in most areas. Marginal effects with pairwise correlations to estimate differences over time are shown. Women living in the Yorkshire and Humber are 3 percentage points less likely to successfully conceive using IVF between 2019-2022 compared to 2009-2018. Whereas married/cohabiting women are 3 percentage points more likely to have a live birth with IVF compared to single women in 2019-2022 compared to 2009-2018. Women living in the middle deprivation quintile (3) are 3 percentage points more likely to have a live birth from IVF in 2019-2022 compared to 2009-2018. However, this was only marginally statistically significant. For the other determinants, none of them are statistically significant suggesting that there had not been a significant change in the influence of these characteristics on the likelihood of having a live birth from IVF after funding declined from 2019.

**Table 3:** Random Effects Probit Model Estimating Likelihood of Live Birth Before and After Reduction in Funding Comparing 2013-2018 to 2019-2022

**Variables**	**Marginal Effects**	**(SE)**
Regions		
North East	‎0.01‎	‎(0.04)‎
North West	‎-0.001‎	‎(0.02)‎
Yorkshire & Humber	‎-0.03**‎	‎(0.01) ‎
East Midlands	‎-0.02‎	‎(0.01)‎
West Midlands	‎-0.01‎	‎(0.02)‎
East of England	‎-0.01‎	‎(0.02)‎
Greater London	Ref.‎	Ref.‎
South East	‎-0.002‎	‎(0.02)‎
South West	‎-0.03‎	‎(0.02)‎
Marital status		
Married/Cohabiting	0.03**	(0.01)
Single	Ref.	Ref.
Urban	-0.01	(0.02)
IMD		
Decile 1	Ref.	Ref.
Decile 2	‎0.01‎	‎(0.01)‎
Decile 3	‎0.03*‎	‎(0.02)‎
Decile 4	‎-0.01‎	‎(0.01)‎
Decile 5	‎0.02‎	‎(0.02)‎
Housing status		
Own house/mortgage	Ref.	Ref.
Social rent	0.001	(0.02)
Private rent	-0.004	(0.02)
Employed	0.004	(0.01)
White British/Irish	0.0001	(0.02)
Education attainment		
Degree or higher	‎-0.03 ‎	‎(0.03) ‎
Some higher education	‎-0.03‎	‎(0.03) ‎
Basic education	‎-0.02‎	‎(0.03)‎
No qualifications	Ref.	Ref.
Observations	6863	

Abbreviation: SE, standard error; IMD, Index of Multiple Deprivation. Notes: Marginal Effects with pairwise correlations are shown. Marginal effects show change in likelihood of live birth for each individual characteristic comparing 2012-2018 to 2019-2022. Standard errors in parentheses. ***P < .01, ** *P* < .05, * *P* < .1.

## Discussion

 In this paper we identify if and how the current provision of IVF in the UK exacerbates regional inequalities in successful birth outcomes from IVF by looking at sociodemographic characteristics of women who successfully conceive using IVF in the UK between 2009-2022 and compare these to women who give birth without using ART. We find that women who live in London, are older, are employed, have a degree-level education or higher, and own or have a mortgage on their home are statistically significantly more likely to have a live birth using IVF. Comparing a period with more generous funding (2009-2018) to a period with less generous funding (2019-2022) we find that in the region with some of the largest cuts for publicly funded IVF such as Yorkshire and Humber, there is a decline in women successfully conceiving using IVF, when funding decreases. Conversely married/cohabiting women and those living in the middle deprivation decline are more likely to conceive using IVF in 2019-2022 compared with 2009-2018.

 Our findings are consistent with much of the literature investigating inequalities in access to IVF treatment that both supply side and demand related issues are likely to create inequities in the health system. It is interesting to note, that this problem exists across different healthcare system designs. Similar findings around the relationship between socio-economic characteristics such as employment status and educational attainment and access to IVF have been found in France and Nordic countries such as Norway, Denmark, and Finland.^11,20-25^ Due to the nature of how IVF is funded in the UK, there have long been concerns of what has been called a “postcode lottery” in access. Jones et al^13^ found that more deprived local authorities had less clinics linking lower levels of funding and resources of people living in some regions to less physical access to clinics. This complements our study which shows that some regions which have higher pockets of deprivation than the national average such as Yorkshire and Humber experienced greater declines in funding. After this decline in funding, people living in this region were less likely to have a live birth using IVF than before the decline.

 Twenty-five years on from the 18th session of the CEDAW outlining women’s right to reproductive and sexual health, many women face barriers to accessing treatment to support their right to reproduce. In light of decreasing birth rates, our results suggest that women with more resources are more likely to benefit from IVF. This has implications for supporting equitable demographic growth and women’s reproductive autonomy.

 Our study has some notable strengths. We use nationally representative longitudinal data from Understanding Society that allows us to study live birth with IVF over 12 years in England. This longitudinal survey includes rich information about women and their families and their socio-economic and socio-demographic status, as well as their region of residence. This builds upon previous work that has relied on administrative data but lacks the contextual information found in household surveys. We also compare live birth with IVF to live birth without IVF, previous studies on IVF pregnancies, compared among women with IVF treatment, those who were successful to those not. We therefore study the determinants of successful conceiving with and without IVF, and overall inequality. The later studies focused on the causal effect of IVF treatment on live birth with IVF among women who had access to and undergo IVF treatment. With our data, we are able to describe what are the factors that are significantly associated with successful live birth with IVF among all women with live birth in the last twelve month and discuss potential driving mechanisms (access, needs, IVF treatment) without being able to disentangle them fully.

 A related potential weakness of our study is that we do not observe the use of IVF or other types of fertility treatment for women who were not successful in getting pregnant. The small proportion of live births with IVF among women having given birth must be acknowledged in our survey sample. Future research should consider linking administrative data which collects precise information on the use of fertility treatment with survey data.

## Conclusions

 Our study finds that some socio-economic characteristics such as employment status, marital status and region of residence are significantly associated with type of conception associated with a live birth. Regional variations in public funding for IVF have been exacerbated by reductions in funding for the health system. This has magnified some of the socio-economic factors associated with conceiving via IVF compared to conceiving naturally. The Women’s Health Strategy for England was launched in 2022 and one of its aims was to improve access to services so that women can meet their reproductive health needs.^32^ However, if the goal of increasing access and ensuring equal access across England is to be achieved, funding will need to be earmarked so that commissioners cannot reduce funding or apply additional criteria for IVF in different Integrated Care Boards.^33^

## Acknowledgements

 We want to thank participants at the ISER seminar series at the University of Essex for their useful feedback on the paper. We want to thank Zuzana Jiraskova for her research assistance with proof reading and editing.

## Disclosure of artificial intelligence (AI) use

 Not applicable.

## Ethical issues

 This study uses secondary data that was downloaded via the UK Data Archive. The University of Essex Ethics Committee has approved all data collection on Understanding Society main study and innovation panel waves.^28^

## Conflicts of interest

 Heather Brown is supported by the National Institute for Health Research Applied Research Collaboration North West Coast (ARC NWC) (NIHR200182) and received travel and accommodation expenses to present this research at the University of Essex.
